# Comparison of Neurofeedback and Transcutaneous Electrical Nerve Stimulation Efficacy on Treatment of Primary Headaches: A Randomized Controlled Clinical Trial

**DOI:** 10.5812/ircmj.17799

**Published:** 2014-08-05

**Authors:** Davood Moshkani Farahani, Seyed Abbas Tavallaie, Khodabakhsh Ahmadi, Ali Fathi Ashtiani

**Affiliations:** 1 Behavioral Sciences Research Center, Baqiyatallah University of Medical Sciences, Teheran, IR Iran

**Keywords:** Primary Headache, Healthcare Providers, Neurofeedback, Transcutaneous Electrical Nerve Stimulation

## Abstract

**Background:**

Headache is one of the most prevalent investigated complaints in the neurology clinics and is the most common pain-related complaint worldwide. Stress is a significant factor that causes and triggers headaches. Since healthcare practitioners experience a lot of stress in their careers, they are more prone to headaches.

**Objectives:**

This study was designed to evaluate and compares the efficacy of neurofeedback behavioural therapy (NFB) and transcutaneous electrical nerve stimulation (TENS) in the treatment of primary headaches in healthcare providers.

**Patients and Methods:**

The current study was a clinical trial, performed in Teheran, IR Iran, with two experimental groups and a control group. Convenient sampling method was used to recruit patients. Independent variables were NFB and TENS and dependent variables were frequency, severity, and duration of headache. Blanchard headache diary was used for assessment. Hence, 45 healthcare providers with primary headache were selected and randomly allocated to one of the NFB, TENS, and control groups by block random assignment method. All three groups completed the headache diary during one week before and after the treatment period as pretest and posttests, respectively. The NFB group was treated in the period between pretest and posttest with fifteen 30-minute treatment sessions three times a week and the TENS group was treated with fifteen 20-minute daily sessions. The control group received none of these treatments.

**Results:**

The results from the analysis of covariance showed that treatment with NFB and TENS had caused significant decrease in the frequency, severity, and duration of headache in experimental groups. The results of the LSD post-hoc test indicated that there were significant differences in the frequency, severity, and duration of pain among experimental groups and the control group. Moreover, there were significant differences between pain frequencies in experimental groups.

**Conclusions:**

According to the results and given the significant reductions in the frequency, severity, and duration of headaches, it seems that NFB and TENS might have an effective role in reducing primary headaches of healthcare providers. In addition, comparing the two methods, treatment with NFB was more effective in reducing headache frequency and severity.

## 1. Backgrounds

Headache is one of the most prevalent problems in the general population and one of the most common complaints at neurology clinics (1). When all types of headaches are considered, headache becomes the most common pain-related complaint worldwide (2). As the most common neurological symptom, it makes painful and debilitating conditions and affects all age groups (3). The overall prevalence of active headache disorders among adult population is 46%; the proportion of tension headaches and migraines is 42% and 11%, respectively (4). Lifetime prevalence of headache in men and woman has been reported as 93% and 99%, respectively (5). Headaches are very important due to disruptions in daily tasks, decreasing working efficiency, and the use of pain-relief medication (6). The high prevalence of this disorder has a very significant effect on job performance as well as quality of life and imposes a great economic burden on society (4, 7).

The most common types of headaches are primary headaches. Primary headaches include migraine headache, tension-type headache, trigeminal autonomic cephalalgia (trigeminal neuralgia) headaches, and other primary headaches such as headaches caused by coughing, exercise, and sexual activity, sinus irritation caused by cold, and headaches caused by direct external tension (8). Migraine headache is chronic and periodic and recurs once in a while (9). Migraine headache has features such as pulsating nature, involving one side of the head, association with nausea, photophobia, and phonophobia, that are debilitating in severe cases. This type of headache usually lasts between four to 72 hours (8).

Psychological stress is considered as the most common trigger and cause of continuation of chronic tension headache attacks. A number of studies have also reported that headaches would continue following the individual’s exposure to psychologically stressful events. Furthermore, patients' usually report that they have tolerated more stress a few days before the headache or simultaneously with it (10, 11).

In a study of 200 Kurdish people in Iraq to determine the alleviating and aggravating factors of migraine headaches, the most common triggers of migraines were stress and psychologic breakdown (12). Another study in Sweden determined that stress played a significant role in inducing migraines (13).

Recent studies also indicate the role of healthcare profession in creating stress for its practitioners. The prevalence of headache directly correlates with the severity of stress that is considerably higher among people with stressful jobs. Healthcare staffs experience job stress, which may lead to serious psychologic and physical health issues (14).

Headache is common among healthcare providers due to high levels of stress. Boran et al. studied occupational stress among 101 physician specialists, 126 dentists, 123 pharmacists, and 52 general practitioners in North Jordan. In their study, 27% of the 402 healthcare providers reported high levels of stress. The prevalence among general practitioners, dentists, and pharmacists was 33%, 30%, and 25%, respectively. The lowest level of stress was reported in physician specialists (12%). The most frequent problems associated with high levels of stress were irritability (58%), consuming more arousal drinks (e.g. coffee or cola) (56%), difficulty in concentrating (51%), headaches (63%), chronic back pain (48%), and common colds (47%) (15).

Neurofeedback behavioral therapy (NFB) is rooted in the belief that the headache is a psycho-physiological disorder. Treatments such as biofeedback and NFB focus on relaxation and physiological responses associated with headache (16).

NFB is the biofeedback based on the brain wave frequency that uses the electrical activity of the brain (electroencephalogram [EEG]) to provide information for the patient. In this context, patients curiously and gradually learn how to modify the electrical activity by data from the electrical activity of the brain (17-20).

Abnormalities in the brainwaves of patients with migraine are demonstrated in previous studies. For example, children with migraines showed increasing theta (θ) waves in comparison with the control group. Thus, NFB can affect the electrical activity of the brain and may be useful for these patients (21-24). Walker investigated the effect of NFB on recurrent migraine headaches on the basis of quantitative EEG (QEEG). The results revealed that headaches stopped in a significant number of participants (54%) and the headaches frequency was reduced by more than 50% in 39% of patients; four patients reported less than 50% decrease in headache frequency and only one patient reported no change in his headache condition (25).

Transcutaneous electrical nerve stimulation (TENS) is also a noninvasive procedure with low risk that can reduce acute and chronic pains (26). TENS is the use of electric current by a specific device to stimulate the nerves for therapeutic purposes. By definition, all areas of the skin are used to stimulate the nerves by electric current. However, this term now usually refers to a limited number of cases of nerve stimulation through the skin, which are usually performed by small and portable devices. These devices are usually attached to the skin by two or more electrodes. In a portable TENS device, bandwidth, frequency, and intensity can be adjusted. Generally, TENS is employed in two forms: high frequency (> 50 Hz) with low intensity or low frequency (< 10 Hz) with high intensity. In the first form, stimulation does not cause response and contraction but it causes motor center response in the second form and inevitably leads to contraction due to high intensity of the stimulation (27). Studies indicate that the mechanism of pain therapy of high-frequency and low-intensity TENS is justified by Melzack and Wall’s gate control theory; in TENS with low frequency with high intensity, the pain therapy relies on the activation and secretion of endogenous opioids system (28-30).

In this context, Mousavi et al. investigated the effects of TENS and imipramine in the prophylaxis of chronic tension-type headache. The investigation concluded that both methods could dramatically reduce the severity of chronic tension-type headache. Therefore, TENS was proposed as a long-term treatment of chronic tension-type headaches (31).

## 2. Objectives

The main purpose of this study was to determine and compare the efficacy of NFB and TENS in the treatment of primary headaches of healthcare providers.

## 3. Patients and Methods

The present study was a clinical trial with two experimental groups and a control group. Convenience sampling method was used to enroll participants. Independent variables were NFB and TENS and dependent variables were frequency, severity, and duration of headache.

### 3.1. Participants

The participants in this study were healthcare providers with primary headaches who were referred to a governmental general hospitals (with 50 wards and over 1000 inpatient bed) and a polyclinic in Tehran, Iran. After interviewing and clinical examination, patients were included according to the international classification of headache disorders-III (ICHD-III, beta version), international headache society (IHS), and the diagnosis of primary headache. By employing simple block random assignment method, 45 patients were randomly assigned to three groups of NFB, TENS, and control. The research was conducted in Teheran, Iran, during the summer of 2013. 

### 3.2. Inclusion Criteria 

According to the criteria of the ICHD-III, we included patients with primary headaches who had developed headaches after entering the health professions. Samples were working as healthcare professional for at least three years. They presented no underlying disease and no abnormalities causing headaches. Subjects were not diagnosed with schizophrenia, major depressive disorder, or addiction. They had no significant trauma to the head and had at least a high school education.

### 3.3. Exclusion Criteria

Patients with the following conditions were excluded: any kind of underlying condition causing headaches, diagnosis of schizophrenia, major depressive disorder, or addiction, receiving other treatments for headache at the time of study, history of a head trauma during the study period, and reluctancy to continue the treatment. In order to measure the parameters of our study, the following tools and methods were used.

### 3.4. A Researchers-Made Headache Questionnaire According to the International Classification of Headache Disorders-III

Researchers structured a questionnaire to precisely estimate the clinical symptoms of primary headache, to determine the headache types, and to collect demographic data. This questionnaire was structured based on the symptoms of headache diagnostic criteria listed in the table of the ICHD-III and was approved by several experts in the field.

### 3.5. Diagnostic Interview According to the Criteria of DSM-V and the International Headache Society 

Diagnostic interview was performed based on the criteria of primary headache disorder in DSM-V and IHS by a psychiatrist.

### 3.6. Blanchard Headache Diary 

This tool has been developed by Blanchard, one of the scientists of headache. Blanchard et al. conducted some studies to determine the validity of this instrument, which is summarized as follows. To determine the reliability of the headache diary, obtained ratings from the significant others of patients with headache were compared to daily diary ratings made by the patients themselves in order to socially validate the headache diary. The correlation between these two measures was significant (r = 0.44). Global ratings that was made by the patients correlated significantly with the diary reports (r = 0.36). These results indicated that the improvement detected by the headache diary, the most common form of self-report assessment in headache research, was significantly perceived by others in the patient's environment (32). This instrument was approved by the IHS and other known international organizations and is commonly applied in various international researches on headache.

### 3.7. The Research Procedure

Based on objective of the study, sample size was calculated with following formula:

n = (Z_1-α/2_ + _Z1-β_)^2^.(S^2^_1_ + S^2^_2_) / (µ_1_ - µ_2_)^2^

Assuming σ^2^_1_ = σ^2^_2_ = σ^2 ^and use of the effect size index, with use of Cohen (1998) formula (d = µ_1_ - µ_2_ / σ), the above formula can be transformed into the following form:

n = 7.84 / 0.64 = 12.25 ≈ 12 = [(1.96 + 0.84)^2^ / 0.8^2^].n = (Z_1-α/2_ + Z_1-β_)^2^ / d^2^

Effect size of 0.8 was considered in calculation. The result was about 12 subjects for each group and we considered 15 participants in each group. Using block random assignment method, 45 subjects were selected from the patients referred to two medical centers in Tehran, Iran. During the sampling period, 120 patients did not meet the eligibility criteria and therefore, were not enrolled. During the intervention period, three subjects discontinued treatment, which were replaced by three new subjects. A psychiatrist and a neurologist examined all of the 45 patients who met the eligibility criteria. Then the subjects completed the diagnostic questionnaire and demographic characteristics and were diagnostically interviewed by the researcher. Participants were randomly assigned to one of the NFB, TENS, and control groups by block random assignment method. After the record of headaches in headache diary (as a pretest) during a week (four times per day), subjects in NFB group received 30-minute treatment sessions three times a week; patients in TENS group underwent 20-minute daily sessions. A total of 15 and 20 sessions were held for patients in NFB and TENS groups, respectively. The control group received none of these treatments. After the treatment period, all groups completed the headache diary for another week (as a post-test).

#### 3.7.1. Neurofeedback

In the NFB group, in each session migraine treatment protocol including suppressing Theta (4-8), strengthening the sensorimotor rhythm (SMR) (12-15 Hz), and suppressing high beta (21-30 Hz) was performed at T3 and T4 sites. Each intervention was performed in a quiet environment; the first five minutes were spent on refreshment and then the basic information of brain waves was recorded for two minutes. Afterwards, migraines protocol was implemented for 30 minutes.

To get the basic data as well as to implement the protocol, electrodes were placed on the subjects’ scalp. These sensors received the brain electrical activities, transferred them to the main unit of NFB device, and transferred them to a computer. After that, the computer simulated brainwaves and displayed them as a computer game or a video to the subjects. In this step, playing the movie or directing the computer game was performed only with subjects’ brainwaves, and thereby the patients could learn to control some of their brain actions. This situation continued throughout the 15 sessions.

#### 3.7.2. Transcutaneous Electrical Nerve Stimulation 

In this study, TENS was performed using a CEFALY-brand machine (STX-Med Sprl factory, Belgium). The device has CE approval in Europe. The device is portable as well as lightweight and is shaped as glasses. After degreasing the skin, the device was installed on the skin of the forehead by special electrodes and transmitted electrical stimulation to the frontal branch of the trigeminal nerve. The necessary bandwidth, frequency, and duration for treatment were adjusted by the company; therefore, no more adjustment was needed. However, the current intensity had to be set according to patients’ tolerance threshold.

The refreshment period was performed in each session. After refreshment and degreasing of the forehead skin with a napkin impregnated with alcohol, the disposable electrodes were placed on the forehead skin. After the final control, device was turned on by the therapist and the intensity was adjusted based on the patient’s threshold. After 20 minutes of treatment, the device was turned off automatically. The electrode was then removed and the site of installation was cleaned. 

### 3.8. Ethical Considerations

In order to comply with ethical principles, the followings were considered: participation in the study was completely voluntary; during the study, the authors did not receive any money from clients; therapy sessions were adjusted according to patients’ time; and patients’ information remained confidential. Approval was obtained from Research Ethics Committee (Code: 383; date: June 12, 2012).

### 3.9. Method of Data Analysis

Data were analyzed with SPSS version 17 (SPSS Inc, Chicago, IL, USA). A Kolmogorov-Smirnov test was used to determine whether the recorded data were distributed normally. Quantitative variables were presented as the mean ± standard deviation (SD) whereas qualitative data were presented as frequency and percentages. Analysis of variance (ANOVA) and analysis of covariance (ANCOVA) were used for continuous variables. To compare and identify differences between groups, LSD post-hoc test was used. Categorical variable were analyzed using Chi square test. In this study, the P value ≤ 0.05 was considered as statistically significance.

## 4. Results

### 4.1. Distribution of Sex, Age, and Education

Demographic data of participants are given in Table 1.

**Table 1.** Demographic Data in the Study Groups ^a,b,c^

Variables	Group			P Value
	NFB	TENS	Control	
**Sex**				0.456
Male	8 (53)	9 (60)	8 (53)	
Female	7 (47)	6 (40)	7 (47)	
**Education**				0.196
Diploma	2 (13)	3 (20)	2 (13)	
Associate Diploma	4 (27)	4 (27)	3 (20)	
Bachelor	4 (27)	2 (13)	4 (27)	
Master's Degree or Higher	5 (33)	6 (40)	6 (40)	
**Age**	37.60 ± 7.462	40.73 ± 10.124	37.33 ± 9.447	0.885

^a^ Continuous variable were analyzed by ANOVA and categorical variable by Chi square test.

^b^ Abbreviation: NFB, neurofeedback behavioral therapy; TENS, transcutaneous electrical nerve stimulation.

^c^ Data are presented as No. (%) and mean ± SD.

### 4.2. Headache frequency

The results of pretest and post-test scores of headache frequency in the NFB, TENS, and control group are shown in Table 2. Before using ANCOVA, Levene test was used to examine the assumption of equality of variances in headache frequency, severity, and duration. The equality of variances of the three groups was confirmed based on the results of the test (P > 0.05). In addition, before using ANCOVA, the assumption of normality was tested by Kolmogorov-Smirnov test.

In order to compare the mean of subjects’ headache frequency, ANCOVA was applied. This test showed statistically significant difference between groups (P < 0.001). The efficacy of these interventions was equal to 30% (Table 3).

**Table 2.** Pretest and Post-Test Scores of Pain Frequency in Study Group ^a^

Group	Pretest	Post-test	P Value
**NFB **			0.001
Minimum	2	0	
Maximum	8	7	
Mean	4.00	2.60	
SD	1.852	1.765	
**TENS**			0.001
Minimum	3	2	
Maximum	10	7	
Mean	5.37	3.33	
SD	2.159	1.676	
**Control**			0.417
Minimum	1	2	
Maximum	8	8	
Mean	4.60	4.43	
SD	1.765	1.534	

^a^ Abbreviations: NFB, neurofeedback behavioral therapy; TENS, transcutaneous electrical nerve stimulation; and SD, standard deviation.

**Table 3.** Analysis of Covariance Results for the Mean Differences of Headache Frequency, Severity, and Duration Scores for Study Groups

Variable	Degree of Freedom	Mean Squares	F Value	P Value	Effect Size	Statistical Power
Pain Frequency						
Pretest	1	62.199	47.517	0.0001	0.537	1.000
Group Membership	2	11.580	8.847	0.001	0.301	0.961
**Pain Severity**						
Pretest	1	68.865	91.317	0.0001	0.690	1.000
Group Membership	2	5.983	7.934	0.001	0.279	0.940
**Pain Duration**						
Pretest	1	1620586.502	43.219	0.0001	0.513	1.000
Group Membership	2	127595.824	3.403	0.043	0.142	0.608

**Figure 1.**
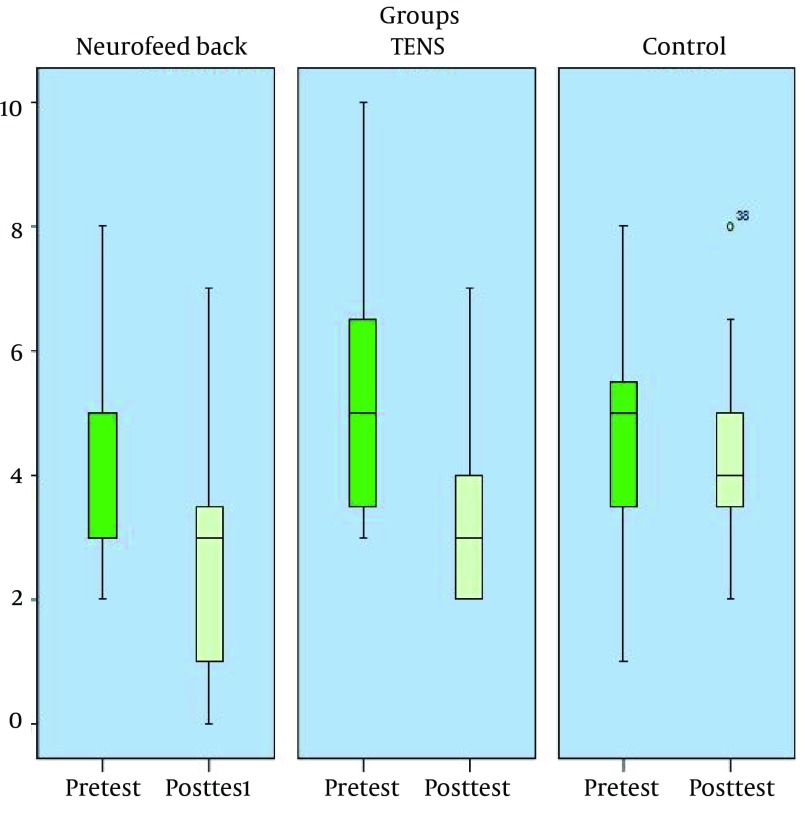
Pretest and Post-Test Mean Score of Pain Frequency in the Experimental and the Control Groups

In order to determine the differences in headache frequency between groups, LSD post-hoc test was used and the results indicated a significant difference between the NFB and the control group (P < 0.01). There was also a significant difference between the TENS and control group (P < 0.05). The difference between the TENS and the NFB groups was also significant (P < 0.05). The pretest and post-test mean scores of pain frequency in study groups are shown in Figure 1.

### 4.3. Headache Severity

The mean of pretest and post-test scores for headache severity in the study groups are shown in Table 4.

**Table 4.** Pretest and Post-Test Scores of Pain Severity in the Study Groups ^a^

Group	Pretest	Post-test	P Value
**NFB **			0.01
Minimum	1.5	0	
Maximum	8	8	
Mean	5.1653	4.1847	
SD	1.6902	1.9798	
**TENS**			0.0001
Minimum	2.70	2.50	
Maximum	7.70	7	
Mean	5.4247	4.5133	
SD	1.4268	1.3517	
**Control**			0.419
Minimum	3.25	3.50	
Maximum	8	8	
Mean	5.5467	5.6667	
SD	1.1788	1.1751	

^a^ Abbreviations: NFB, neurofeedback behavioral therapy; TENS, transcutaneous electrical nerve stimulation; and SD, standard deviation.

ANCOVA was used to compare the mean of headache severity among study groups. This test showed statistically significant difference between groups (P < 0.001). The efficacy of this interventions was equal to 28% (Table 3).

**Figure 2.**
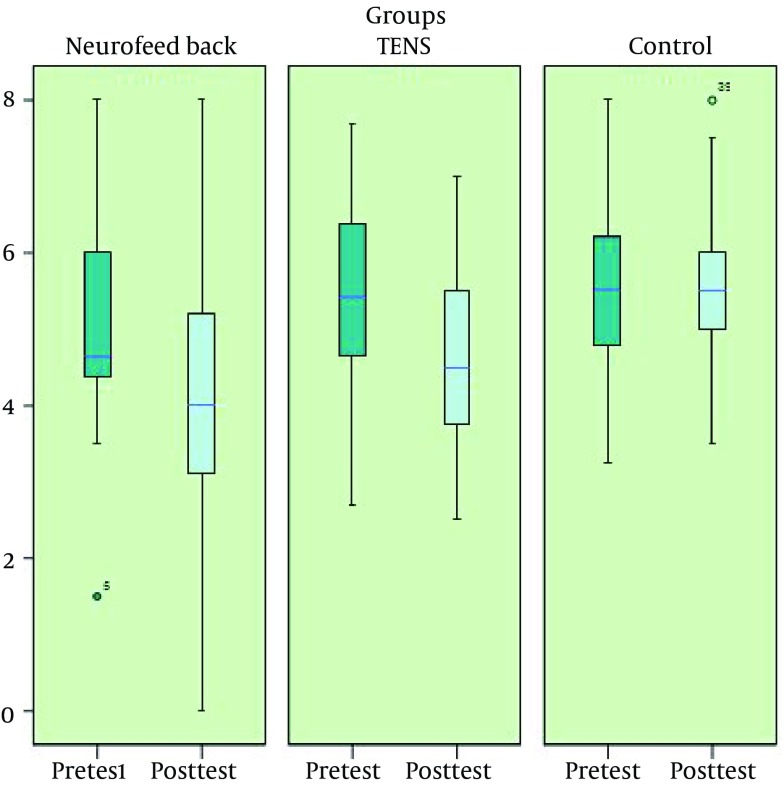
Pretest and Post-Test Mean Score of Pain Severity in the Study Groups

In order to determine the differences between the groups, LSD post-hoc test was used and the results indicated a significant difference between the NFB and the control groups (P < 0.01) as well as between the TENS and the control groups (P < 0.05); however, the difference between the NFB group and TENS group was insignificant. The pretest and post-test mean scores of pain severity in the study groups are shown in Figure 2.

### 4.4. Headache duration

The pretest and post-test scores of headache duration in the study group are shown in Table 5.

**Table 5.** Pretest and Post-Test Scores of Pain Duration in the Study Groups ^a^

Group	Pre-test	Post-test	P Value
**NFB **			0.05
Minimum	120	0	
Maximum	1440	1440	
Mean	555.13	357.47	
SD	429.784	376.205	
**TENS **			0.01
Minimum	102	80	
Maximum	493	840	
Mean	454.80	343.27	
SD	263.627	197.065	
**Control**			0.465
Minimum	160	160	
Maximum	880	860	
Mean	456.00	452.00	
SD	204.548	197.708	

^a^ Abbreviations: NFB, neurofeedback behavioral therapy; TENS, transcutaneous electrical nerve stimulation; and SD, standard deviation.

ANCOVA was used to compare the mean of subject’s headache duration. This test showed that the difference between groups was statistically significant (P < 0.05). The efficacy of this intervention was equal to 14% (Table 3).

**Figure 3.**
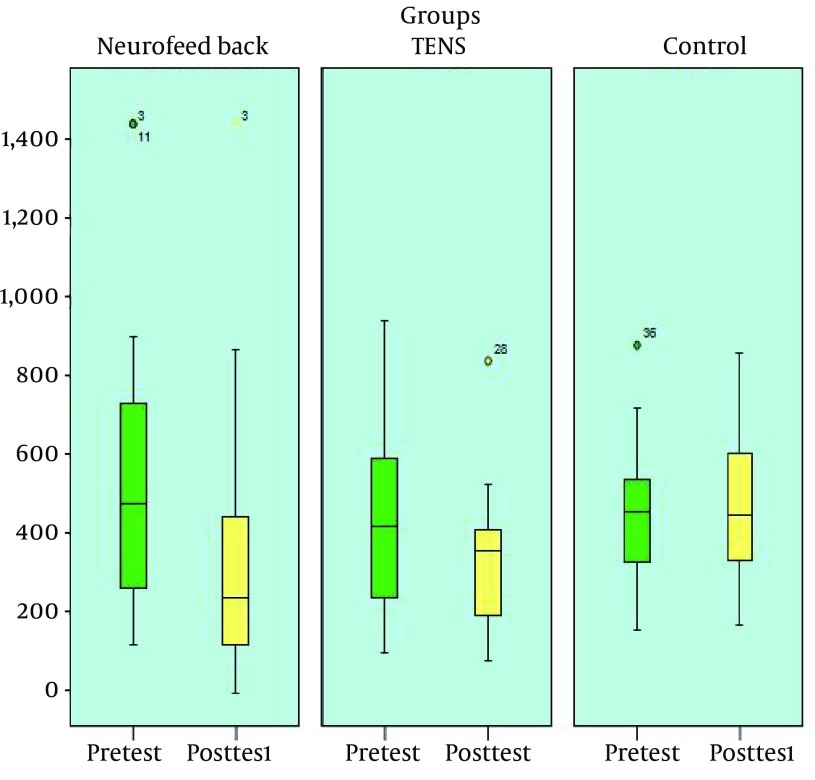
Pretest and Post-Test Mean Score of Pain Duration in the Study Groups

LSD post-hoc test was used to determine the differences between the study groups and the results indicated a significant difference between the NFB and the control groups (P < 0.05) as well as between TENS and control groups (P < 0.05); however, the difference between the NFB and TENS groups was insignificant. The pretest and post-test mean scores of pain duration in the study groups are shown in Figure 3.

## 5. Discussion

The aim of this study was to compare the efficacy of NFB and TENS in the treatment of primary headaches in healthcare providers. The results indicated a significant difference between the pretest and post-test mean scores of frequency, severity, and duration of primary headache in the experimental and control groups. Moreover, since the NFB and TENS were performed in the interval between the pretest and the post-test, it could be concluded that NFB and TENS therapeutic interventions were effective in the treatment of primary headache.

In examining the differences between study groups and comparing their efficiency, there was a significant differences between the experimental groups (NFB and TENS) and the control group with regard to the headache frequency. Moreover, there was a significant difference between the NFB and TENS groups and NFB group showed a greater reduction in headache frequency in comparison to TENS group. 

In addition, there was a significant differences between experimental groups (NFB and TENS) and the control group with regard to the severity of headache. Although the difference between NFB and TENS was insignificant, the reduction of headache severity was greater in the NFB group in comparison with TENS group. There was a significant differences between the experimental groups (NFB and TENS) and the control group regarding the headache duration. Although the difference between the NFB and TENS group was insignificant, the reduction in the mean score of headache duration was greater in TENS group in comparison with NFB group.

The results in the NFB group were consistent with the Stokes and Lappin's study that aimed to evaluate the efficacy of NFB and thermal biofeedback in the treatment of migraine headaches. A total of 37 migraineurs underwent 40 sessions of NFB in combination with thermal biofeedback and finally, 27 patients (70%) reported more than 50% reduction in the frequency of headache, which remained the same on average 14.5 months after discontinuation of the treatments (33).

These results in TENS group were also in line with Schoenen et al. study that aimed to assess the efficacy and safety of trigeminal electrical nerve stimulation by supraorbital transcutaneous stimulator in order to treat migraine headaches. In this double-blinded study, 59 migraineurs were studied. In the first month of intervention, the number of days with migraine in both active and dummy groups was decreased by 20%; in addition, the number of days with migraine was decrease by 29.7% in the active and by 4.9% in the dummy group in the second and third months (34).

Nonmedical therapies have attracted more attention during the recent decades. Although medications are commonly used to treat headaches, they are inefficient, inadequate, and inappropriate for a significant number of patients. Due to the low tolerance to the drugs, achieving unfavorable response, and the side effects of tranquilizers/sedatives in long term, nonmedical approaches are sought to treat headaches.

Through treatment with NFB and TENS, a person would not need drug. Moreover, these methods are noninvasive, harmless, portable, and relatively inexpensive and do not interfere with other treatments. In addition, they are even available during the work shifts. According to current statistics and considering the high incidence of headache among healthcare practitioners and a correlation between tension headaches and migraines with healthcare profession, NFB and TENS can be very useful and increase the efficiency of healthcare practitioners. NFB has better efficacy than TENS in decreasing frequency and severity of headaches and is more noninvasive.

### 5.1. Strengths

1. This study was conducted on healthcare providers with primary headaches; such a study had not been done before.
2. This study was conducted in the form of clinical trial.
3. In this study, two treatment methods with two different mechanisms were compared, which provides a good guideline for further studies.
4. The study revealed a more effective and more persistent treatment.

### 5.2. Weaknesses

Small sample size and lack of random sampling were the weaknesses of this study.
